# Fluoroquinolone-Induced Metabolic Dysregulation and Oxidative Stress Orchestrate Bacterial Demise

**DOI:** 10.3390/microorganisms14051108

**Published:** 2026-05-13

**Authors:** Caiyuan Zhou, Jing Sun, Yihan Luo, Fang Wang, Luqi Li, Tong Wu, Peng Xie, Chenxi Liu, Yibin Hu, Leilei Sun, Chengbao Wang

**Affiliations:** 1 College of Veterinary Medicine, Northwest A&F University, Yangling, Xianyang 712100, China; 2 Life Science Research Core Services, Northwest A&F University, Yangling, Xianyang 712100, China

**Keywords:** fluoroquinolones, metabolomics, oxidative stress, reactive oxygen species, *Escherichia coli*, metabolic reprogramming, nucleic acid damage

## Abstract

The bactericidal mechanisms of fluoroquinolones extend beyond their canonical inhibition of DNA topoisomerases, yet the associated metabolic perturbations remain incompletely understood. In this study, we systematically investigated the metabolic responses of *Escherichia coli* to three representative FQs—ofloxacin, enrofloxacin, and ciprofloxacin—using untargeted UPLC–Q Exactive Orbitrap–MS-based metabolomics. Bacterial cells were exposed to bactericidal concentrations (2 × MIC) for a single-time point (1 h), followed by comprehensive metabolomic profiling with six biological replicates per group. Our findings demonstrate that FQ-induced metabolic reprogramming serves as a primary driver of oxidative stress and nucleic acid damage, rather than a mere secondary effect. All three FQs induced substantial metabolic reprogramming characterized by disruptions in nucleotide biosynthesis, central carbon metabolism, and redox-related pathways, with notable drug-specific differences. Ciprofloxacin exhibited the most pronounced suppression of energy metabolism and antioxidant systems, whereas ofloxacin and enrofloxacin showed partial compensatory metabolic responses. Consistently, intracellular ROS levels were significantly elevated in all treatment groups, and this effect was attenuated by antioxidant supplementation. Furthermore, increased accumulation of 8-hydroxydeoxyguanosine and 8-hydroxyguanosine confirmed the occurrence of oxidative DNA and RNA damage. Collectively, these findings indicate that FQs induce distinct metabolic perturbations that are closely associated with oxidative stress and nucleic acid damage, providing a metabolic perspective on their bactericidal activity and suggesting potential targets for metabolic adjuvant strategies.

## 1. Introduction

Using *Escherichia coli* as a primary model organism, the escalating crisis of antimicrobial resistance (AMR) necessitates a fundamental re-evaluation of antibiotic lethality [1,2,3]. For decades, traditional models of bactericidal action have largely focused on drugs as direct inhibitors of specific molecular targets—in the case of fluoroquinolones (FQs), the disruption of type II topoisomerases (DNA gyrase and topoisomerase IV) to generate cytotoxic double-strand breaks [4,5]. However, the sole emphasis on direct target inhibition often falls short in explaining the complex kinetics of bacterial cell death [6,7]. Emerging evidence now posits that the primary drug-target interaction functions as a mere trigger for a systemic bioenergetic catastrophe. This catastrophic cascade culminates in hyperactive cellular respiration, destabilization of iron-sulfur clusters, and rampant generation of reactive oxygen species (ROS), leading to widespread damage to macromolecules [8,9,10]. While the specific contribution of ROS to antibiotic lethality remains a subject of academic debate, elucidating the metabolic context of antibiotic action is paramount, complementing and refining our understanding of their primary target interactions.

Unraveling these terminal pathologies necessitates a shift from analyzing genomic potential to characterizing phenotypic execution, a task for which untargeted metabolomics is uniquely poised [11,12]. In contrast to transcriptional data, which often exhibits an imperfect correlation with protein function, the metabolome offers one of the most direct functional readouts of cellular state—a real-time chemical portrait of physiology under duress [13,14]. By capturing the instantaneous fluctuations of small molecules, metabolomics can delineate genuine survival mechanisms from mere metabolic perturbations [15,16]. This distinction holds particular relevance for fluoroquinolones (FQs), which impose a distinct metabolic dilemma on bacteria: FQs activate an energetically demanding SOS response for DNA repair, yet the very metabolic activity required to fuel this repair may inadvertently exacerbate ROS production, thereby potentiating lethality [17]. Consequently, the pre-existing and drug-induced metabolic state acts as a critical determinant, dictating whether a bacterium successfully navigates initial DNA damage or ultimately succumbs to systemic metabolic collapse [18].

Central to this metabolic perspective is the intricate interplay between the tricarboxylic acid (TCA) cycle and antibiotic efficacy [19]. Recent work has refined the long-held assumption that faster growth invariably correlates with higher susceptibility, revealing instead that basal metabolic rate and electron transport chain efficiency are primary determinants of drug efficacy [20]. Bacteria frequently evade FQ-induced lethality not solely via genetic mutation, but through adaptive metabolic strategies such as ‘metabolic dormancy’—downregulating the TCA cycle to mitigate respiration-linked damage—or by rerouting carbon flux via the glyoxylate shunt [21]. Conversely, enhancing TCA cycle activity can trap bacteria in a ‘futile cycle’ of ATP synthesis and hydrolysis, thereby sensitizing even recalcitrant persister subpopulations [22]. Thus, precisely mapping the FQ-induced alterations in carbon flux extends beyond a descriptive exercise, representing a crucial step towards identifying exploitable metabolic vulnerabilities for therapeutic intervention.

The translational implications of such a metabolic atlas are profound. Identifying specific metabolic pathways that buffer FQ toxicity opens avenues for designing ‘metabolic adjuvants’—non-toxic metabolites capable of reprogramming the bacterial physiological state to potentiate antibiotic killing when co-administered [23]. For instance, supplementation with specific amino acids or sugars has been demonstrated to stimulate proton motive force and facilitate drug uptake in antibiotic-tolerant cells [24,25]. However, a critical unresolved question persists: whether the entire class of fluoroquinolones elicits a monolithic metabolic signature, or if subtle structural distinctions among different generations of these drugs drive divergent physiological responses [26,27]. Given the widespread clinical reliance on FQs across human medicine and veterinary applications, resolving these nuances is paramount for informing precision antibiotic stewardship.

Here, we present a comprehensive investigation aimed at elucidating both class-wide and drug-specific metabolic perturbations induced by fluoroquinolones. Utilizing untargeted UPLC–Q Exactive Orbitrap–MS, we analyzed the temporal metabolic response of *Escherichia coli* to three clinically prevalent FQs: Ofloxacin, Enrofloxacin, and Ciprofloxacin. While these agents share the core mechanism of DNA topoisomerase inhibition, we hypothesized that their distinct physicochemical properties would nonetheless elicit unique compensatory metabolic rewiring responses. Our data reveal a complex metabolic landscape where all three FQs induce a signature disruption of nucleotide biosynthesis and oxidative stress markers, yet exhibit significant divergence in their modulation of amino acid metabolism and central carbon energy flux. These findings collectively establish a comprehensive metabolic atlas of fluoroquinolone toxicity, thereby offering novel targets for adjuvant development aimed at restoring the efficacy of this critical antibiotic class.

## 2. Materials and Methods

### 2.1. Bacterial Strains and Reagents

*E. coli* MG1655 was used for the studies. Bacterial strains were cultivated under the conditions of Luria–Bertani (LB) medium at a temperature of 37 °C. OFLX, ENR, CPFX and *N*-acetylcysteine were purchased from Solarbio (Beijing Solarbio Science & Technology Co., Ltd., Beijing, China). The ROS Detection Assay Kit was purchased from GENMED Scientifics Inc., Framingham, MA, USA. Methanol, acetonitrile, formic acid, and ethanol were sourced from Fisher Chemical (Thermo Fisher Scientific, Waltham, MA, USA). Minimum inhibitory concentrations (MICs) were determined using the broth microdilution method in accordance with Clinical and Laboratory Standards Institute (CLSI) guidelines. For all assays, six independent biological replicates were performed to ensure reproducibility.

### 2.2. SEM Analysis

Bacterial cultures of *E. coli* MG1655 were prepared and stimulated with OFLX (2 μg/mL), ENR (1 μg/mL), and CPFX (1 μg/mL) at an OD600 of approximately 0.30. The cells were then harvested through centrifugation, washed with PBS, and subsequently resuspended in a 3% glutaraldehyde solution for fixation purposes. After fixation for a duration of 1 to 2 h in a 1% osmium tetroxide solution, the samples underwent three consecutive washes with ultrapure water lasting for 10 min each. Dehydration was conducted using a sequential series of ethanol solutions: 30%, 50%, 70%, 90%, and three repetitions of 100% concentration, with each step lasting for a duration of 15 min. Subsequently, the samples were deposited onto silicon wafers via pipetting and affixed to a sample stage using conductive adhesive. The mounted samples were then coated with gold through ion sputtering utilizing an advanced sputtering instrument. Imaging analysis was performed employing the JSM-IT700HR scanning electron microscope (JEOL, Tokyo, Japan). Initially, low magnification observations were made to evaluate the overall morphology of each sample, followed by high-resolution imaging targeting specific regions in order to identify distinct bacterial morphological changes.

### 2.3. Untargeted Metabolomics Analysis

Bacterial cultures of *E. coli* MG1655 were prepared and stimulated with OFLX (2 μg/mL), ENR (1 μg/mL), and CPFX (1 μg/mL) at an OD600 of approximately 0.30 for 1 h. The cultures were quenched using a methanol/ethylene glycol mixture at −60 °C and subsequently washed with a 0.85% NaCl solution. Following this, cell pellets were extracted with boiling ethanol/water (75:25, *v*/*v*) at 95 °C. Metabolite separation was executed through both reversed-phase (RP) and hydrophilic interaction liquid chromatography (HILIC), with detection performed using a UPLC—Q Exactive Orbitrap—MS system (Thermo Fisher Scientific, Waltham, MA, USA) with a mass accuracy of <5 ppm and a resolution of 30,000 FWHM in both positive and negative electrospray ionization modes. For RP separation, a BEH Shield RP C18 column (2.1 mm × 100 mm, 1.7 μm; Waters, Milford, MA, USA) was utilized, employing mobile phases consisting of solvent A (0.1% formic acid in water) and solvent B (0.1% formic acid in acetonitrile) for ESI+ mode, and solvent A (5 mM ammonium acetate in water) with solvent B (5 mM ammonium acetate in acetonitrile) for ESI− mode. The gradient for this separation was as follows: from 0 to 1 min, 2% B; from 2 to 10 min, 2% to 40% B; from 10 to 11 min, 40% to 98% B; from 11 to 12 min, maintaining at 98% B; from 12 to 12.1 min, returning from 98% to 2% B; and from 12.1 to 15 min, at 2% B. HILIC separation was performed using a BEH Amide column (100 mm × 2.1 mm, 1.7 μm; Waters, Milford, MA, USA). In this case, solvent A consisted of 0.1% formic acid plus 5 mM ammonium acetate in water, while solvent B was 0.1% formic acid in acetonitrile for ESI+ mode, and solvent A (5 mM ammonium acetate in water) with solvent B (5 mM ammonium acetate in acetonitrile) for ESI− mode. The linear gradient program for HILIC was as follows: from 0 to 2 min, 95% B; from 2 to 8 min, decreasing from 95% to 70% B; from 8 to 9 min, 70% to 50% B; from 9 to 10 min, maintaining at 50% B; from 10 to 10.1 min, increasing from 50% to 95% B; and from 10.1 to 15 min, remaining at 95% B. The flow rate was set at 0.3 mL/min with an injection volume of 2 μL. The Q Exactive Orbitrap–MS was operated in both positive and negative electrospray ionization modes. Full-scan MS data were acquired at a resolution of 30,000 FWHM over a mass range of *m*/*z* 50–1200. Data-dependent MS/MS acquisition was performed using stepped normalized collision energies of 20, 35, and 50 eV. Instrument parameters, including spray voltage, capillary temperature, sheath gas flow rate, auxiliary gas flow rate, and S-lens RF level, were optimized according to the manufacturer’s recommendations to ensure stable ionization and reproducible signal acquisition. Data were acquired in data-dependent acquisition mode. Analytical validation and quality control. Prior to each batch, the Q Exactive Orbitrap–MS was calibrated using Pierce ESI Positive and Negative Ion Calibration Solutions (Thermo Fisher Scientific, Waltham, MA, USA) to ensure mass accuracy < 5 ppm. Quality control (QC) samples were prepared by pooling equal aliquots of each sample and analyzed every 10 injections to monitor instrument stability and retention time drift; metabolite features with relative standard deviation (RSD) > 30% across QC replicates were excluded from downstream analysis. Metabolite annotation was performed at MSI confidence Level 2 (putatively annotated compounds) by matching accurate mass (<5 ppm), isotopic pattern, and data-dependent MS/MS fragmentation against public databases (HMDB, KEGG, and MassBank). Data processing was carried out using Progenesis QI software (version 2.4; Waters, Newcastle upon Tyne, UK). For metabolomics data, structural identification of metabolites was performed by matching retention times and accurate mass weights with the database. The raw data were normalized by total ion current. Differential metabolites were screened using a combination of VIP values (VIP > 1.0) and *p*-values (*p* < 0.05). False discovery rate (FDR) correction was applied using the Benjamini–Hochberg method to minimize false positives. Authentic standards for key energy metabolites (e.g., glutathione, acetyl-CoA, FAD, ATP) are available in our laboratory for subsequent targeted validation.

### 2.4. Determination of Intracellular ROS Accumulation

Individual colonies of *E. coli* MG1655 were carefully isolated and subsequently inoculated into sterile LB broth for overnight culture at 37 °C. The overnight cultures were then transferred to conical flasks containing fresh LB broth and grown until the optical density (OD600) reached approximately 0.30. Following this, the cultures were centrifuged at 8000 rpm for 5 min and washed twice with 30 mL of sterile saline solution. The resulting pellet was resuspended in M9 minimal medium enriched with ammonium acetate (10 mM), MgSO_4_ (1 mM), and CaCl_2_ (100 μM). M9 medium was selected instead of LB to minimize the interference of complex medium components with fluorescence signals and to prevent the quenching of ROS-sensitive probes. To these resuspended cultures, OFLX at 2 μg/mL, ENR at 1 μg/mL, and CPFX at 1 μg/mL were added, along with 10 mM hydrogen peroxide (H_2_O_2_) as the positive control. Each treatment group was set up in triplicate and incubated at 37 °C for 1 h. Intracellular reactive oxygen species (ROS) levels were quantified using a High-Quality Fluorescent Assay Kit (GENMED Scientifics Inc., Framingham, MA, USA) and measured with a Multi-mode Microplate Reader (BioTek, Winooski, VT, USA) at excitation/emission wavelengths of 485/535 nm. Results are expressed in Relative Fluorescence Units (RFU) normalized to the control group.

### 2.5. Inhibition of Intracellular ROS

*E. coli* MG1655 was adjusted to achieve a turbidity corresponding to a McFarland standard of 0.5 and subsequently diluted in 10 mL of M9 media at a 1:100 ratio, resulting in an estimated concentration of 10^6^ CFU/mL. To the diluted cultures, OFLX was added at a concentration of 2 μg/mL, ENR at 1 μg/mL, and CPFX at 1 μg/mL, with 10 mM hydrogen peroxide (H_2_O_2_) serving as the positive control. Furthermore, *N*-acetylcysteine was included in the antibiotic treatment group as a ROS inhibitor at a concentration of 10 mM. ROS levels were measured after an incubation period of 1 h.

### 2.6. ROS-Induced Nucleic Acid Damage

Overnight cultures were diluted at a ratio of 1:250 in 25 mL of LB medium contained within 250 mL baffled flasks and incubated until an OD600 of 0.30 was reached. Subsequently, the cells underwent treatment with OFLX (2 μg/mL), ENR (1 μg/mL), CPFX (1 μg/mL), and H_2_O_2_ (10 mM) for each specific group for a duration of 1 h. Following treatment, the cells were harvested and subjected to centrifugation at 4000 rpm for 10 min using a benchtop swinging-bucket centrifuge. After washing with PBS, the resulting pellets were resuspended in 400 µL of 1% SDS dissolved in dH_2_O. The resuspended samples were transferred into Lysing Matrix B tubes (MP Biomedicals, Irvine, CA, USA))and vortexed three times for 45 s each, allowing the samples to cool on ice between vortexing periods. RNA extraction was performed utilizing a phenol-chloroform method, while DNA purification was achieved with the QIAmp DNA Mini kit (Qiagen, Hilden, Germany). The quantification of 8-OHdG was carried out using the OxiSelect Oxidative DNA Damage ELISA kit (Cell Biolabs, San Diego, CA, USA), and the assessment of 8-OHG levels was performed with the OxiSelect Oxidative RNA Damage ELISA kit (Cell Biolabs, San Diego, CA, USA). Each sample underwent analysis in six replicates.

### 2.7. Statistical Analysis

Statistical analysis was conducted using unpaired two-sided Student’s *t*-tests or one-way ANOVA to evaluate significance. Multiple comparisons following ANOVA were analyzed using Tukey’s post hoc test. For metabolomics data, *p*-values were explicitly adjusted using the Benjamini–Hochberg False Discovery Rate (FDR) method, with significance set at *q* < 0.05. The significance levels in figures were defined as follows: * *p* < 0.05, ** *p* < 0.01.

## 3. Results

### 3.1. Defining Fluoroquinolone Concentrations That Induce Bacterial Metabolic Perturbation

**Figure 1:**
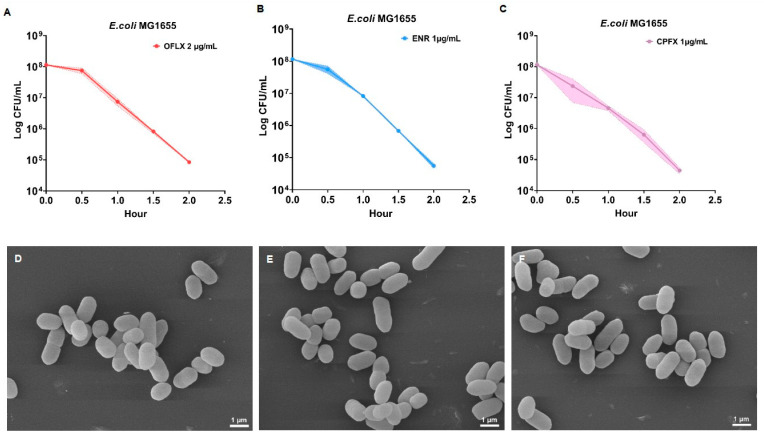
Selection of concentrations that perturb bacterial metabolism via Fluoroquinolone antibiotics. (**A**–**C**) The growth curve of *E. coli* stimulated by the presence of OFLX, ENR, and CPFX. (**D**) OFLX stimulates *E. coli*, 10,000×. (**E**) ENR stimulates *E. coli*, 10,000×. (**F**) CPFX stimulates *E. coli*, 10,000×. Data are presented as mean ± SD (n = 6).

To establish the antimicrobial efficacy of fluoroquinolones under controlled laboratory conditions, we first determined the bactericidal activity of three selected fluoroquinolone antibiotics against *Escherichia coli* strain MG1655 using time-kill kinetic assays. The minimum inhibitory concentrations (MICs) of OFLX, ENR, and CPFX against *E. coli* MG1655 were determined to be 1 μg/mL, 0.5 μg/mL, and 0.5 μg/mL, respectively, according to CLSI guidelines. Each drug was subsequently tested at a concentration equivalent to 2-fold its respective MIC (OFLX: 2 μg/mL; ENR: 1 μg/mL; CPFX: 1 μg/mL). As depicted in Figure 1A–C, all three fluoroquinolones exhibited potent and rapid bactericidal effects. Within 1.5 h of antibiotic exposure, the viable cell count of *E. coli* MG1655 consistently decreased by more than three orders of magnitude across all treatments. To further characterize the cellular impact at these bactericidal concentrations and to ensure the suitability of these conditions for subsequent metabolomic investigations, we performed scanning electron microscopy (SEM) analyses of *E. coli* MG1655 cells treated with each fluoroquinolone. Critically, Figure 1D–F demonstrates that at the tested concentrations, bacterial cells maintained their intact morphology, showing no overt signs of membrane damage or lysis. This morphological integrity indicates that the bacterial cell envelope remains physically intact under these bactericidal concentrations. While SEM cannot directly prove metabolic integrity, the absence of visible membrane disruption supports that observed metabolite changes reflect active cellular responses rather than non-specific passive leakage.

### 3.2. Fluoroquinolones Induce Distinct Metabolic Reconfigurations

**Figure 2:**
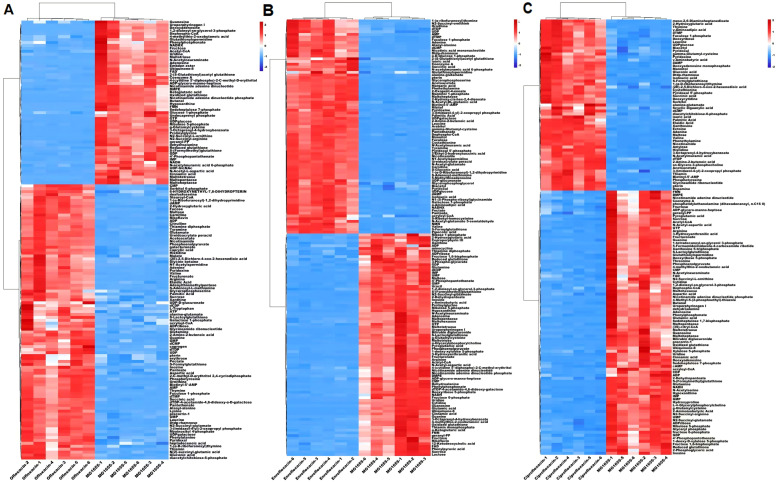
Differential metabolic profile mediated by Fluoroquinolone antibiotics. (**A**) Differential metabolites after stimulation of *E. coli* by OFLX. (**B**) Differential metabolites after stimulation of *E. coli* by ENR. (**C**) Differential metabolites after stimulation of *E. coli* by CPFX. Differential metabolites were selected based on VIP > 1.0 and *p* < 0.05 (FDR-corrected).

This study meticulously characterized the metabolic impact of three distinct fluoroquinolone antibiotics-ofloxacin (OFLX), enrofloxacin (ENR), and ciprofloxacin (CPFX)-on *E. coli*. Utilizing UPLC-Q-Exactive Orbitrap-MS, we uncovered specific, yet often divergent, perturbations in the intracellular metabolome of bacteria challenged with these agents. To delineate the unique metabolic signatures induced by each fluoroquinolone, we performed hierarchical clustering analysis on the differentially abundant metabolites identified across treated and control *E. coli* samples. The resulting heatmaps (Figure 2) provide a high-resolution visualization of these metabolic shifts; columns denote individual samples, while rows correspond to distinct metabolites, with the accompanying dendrogram illustrating the inherent clustering relationships among treatment groups.

Metabolomic assessment through heatmap clustering analysis revealed a substantial and multifaceted influence of OFLX treatment on the *E. coli* metabolome (Figure 2A). Across the 12 analyzed samples, encompassing both OFLX-treated and control groups, we identified 150 differentially regulated metabolites; notably, 94 exhibited elevated levels, while 56 were diminished. Upregulated metabolites spanned diverse metabolic categories, including crucial coenzymes such as propionyl-CoA, palmitoyl-CoA, and pantothenate, indicating potential compensatory or perturbed coenzyme synthesis. Polyamine metabolism was profoundly altered, marked by a decrease in glutathionylspermidine alongside an increase in S-adenosyl-L-methionine, suggesting a dysregulation of cellular growth and stress responses. Furthermore, a significant elevation was observed in amino acid-related metabolites, particularly glutamate, serine, alanine-glutamate, and leucine, pointing towards altered protein turnover or amino acid biosynthesis pathways. Metabolites associated with phenylalanine metabolism (including tyrosine and acetoacetate) and fatty acid metabolism were similarly upregulated. Nucleotide metabolism displayed heightened levels of UMP, ADP-ribose, pterin, inosine, xanthine, guanine, aspartate, and adenine, alongside carbohydrate metabolism indicators such as maltose, reflecting complex shifts in nucleic acid synthesis and energy substrate utilization. Conversely, the downregulated metabolites primarily pertained to core energy metabolism, encompassing critical components like acetyl-CoA, reduced glutathione, GTP, oxidized glutathione, NADH, and NADHX. We also noted a reduction in metabolites vital for cellular structural synthesis, such as UDP-glucose, GTP, and acetyl-CoA. Antioxidant and stress response pathways were likewise compromised, evidenced by the decrease in both reduced and oxidized glutathione. Other downregulated metabolites included those linked to sugar metabolism, like alpha-ketoglutarate, and nucleotide/nucleic acid metabolism, such as adenosine.

Our metabolomic analysis of ENR-treated *E. coli* revealed distinct concentration changes in 161 metabolites, with 79 being upregulated and 82 downregulated (Figure 2B). Among the upregulated metabolites, we identified essential cofactors and coenzymes, including dephospho-CoA, propionyl-CoA, and nicotinamide, suggesting an adaptive response to maintain cofactor pools. Additionally, metabolites associated with amino acid metabolism and biosynthesis, such as leucine, isoleucine, and γ-glutamylcysteine, exhibited significantly elevated levels, indicating altered protein synthesis or degradation. Intriguingly, polyamine metabolism displayed an upregulation of glutathionylspermidine and N1-acetylspermidine, contrasting with OFLX and potentially signifying different stress coping mechanisms. Lipid metabolism also showed increases in palmitic acid, glycerophosphoglycerol, and glycerophosphoserine. Phenylalanine metabolism intermediates, such as acetoacetate, were similarly upregulated. In striking contrast, the downregulated metabolites were predominantly related to central energy metabolism, including markedly decreased levels of acetyl-CoA and ADP. Furthermore, compounds pertinent to nucleotide metabolism (e.g., uridine monophosphate, xanthine, and hypoxanthine) also decreased. Compounds indispensable for cellular structural synthesis, such as the chromatin component acetyl-CoA and the biofilm component dimethylphosphatidylethanolamine, were notably reduced. The analysis further highlighted the downregulation of antioxidant and stress response pathways, as well as alterations in signaling pathways, carbohydrate metabolism, and nucleotide/nucleic acid metabolism.

Metabolomic profiling of CPFX-treated *E. coli* exposed significant concentration shifts in 145 metabolites, with 57 showing upregulation and 88 downregulation (Figure 2C). The upregulated metabolites were largely devoid of the pronounced coenzyme and cofactor increases seen with OFLX and ENR, primarily comprising lipid-related compounds like palmitic acid and choline metabolism-associated compounds such as sn-glycerol-3-phosphocholine. Conversely, the downregulated metabolites predominantly impacted crucial aspects of energy metabolism (e.g., GTP, ADP, and propionyl-CoA), including various coenzymes or cofactors such as coenzyme A, acetyl-CoA, dephospho-CoA, and NADH, pointing to a widespread energy deficit. Furthermore, we observed reduced levels of amino acid metabolism and biosynthesis-related metabolites, including acetyl-CoA, glutamate, glutamine, and reduced glutathione, as well as compounds linked to nucleotide metabolism (hypoxanthine, guanosine, and cytidine). Phenylalanine metabolism-associated compounds (e.g., cinnamic acid) were similarly decreased. Additionally, metabolites involved in carbohydrate metabolism, such as xylulose-5-phosphate, fructose-1,6-bisphosphate, fructose-6-phosphate, and ribulose-5-phosphate, exhibited diminished levels, indicative of impaired central carbon metabolism. Crucially, antioxidant compounds and those participating in stress responses, including both reduced and oxidized glutathione, were also significantly downregulated. Collectively, CPFX treatment induced a broad spectrum of metabolic changes in *E. coli*, characterized by a marked reduction in energy metabolism coupled with severely attenuated stress response and antioxidant capacities. 

### 3.3. Divergent Metabolic Reprogramming Drives Fluoroquinolone Bactericidal Action

**Figure 3:**
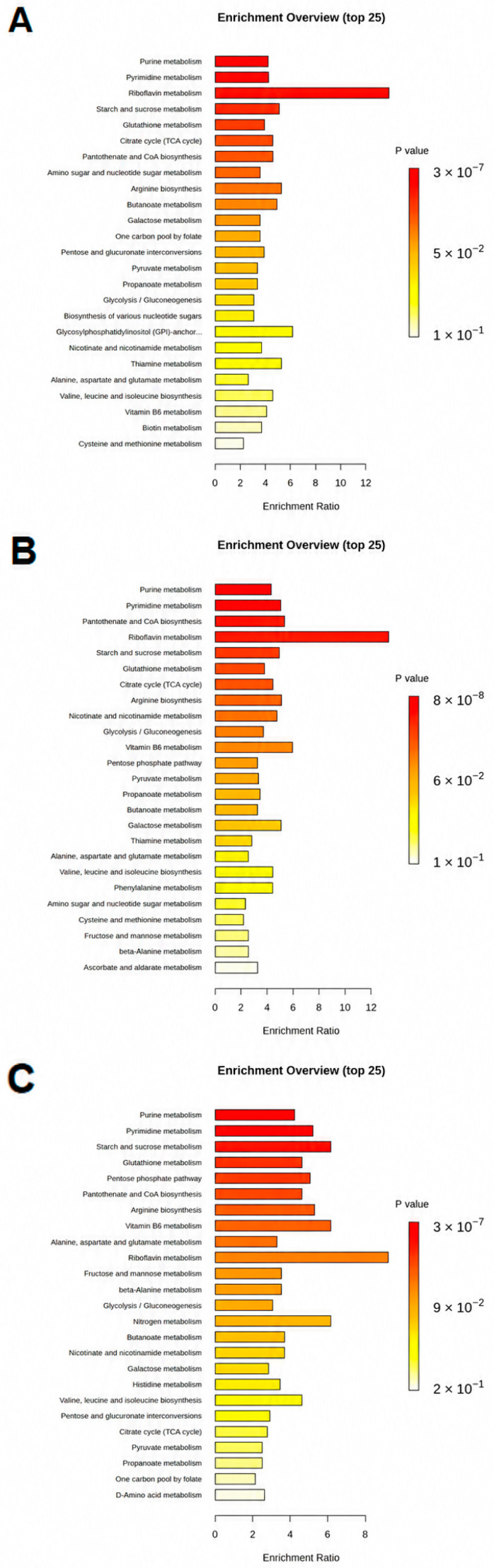
Enrichment analysis of differential metabolites. (**A**) Enrichment analysis of differential metabolites in *E. coli* stimulated by OFLX. (**B**) Enrichment analysis of differential metabolites in *E. coli.* stimulated by ENR. (**C**) Enrichment analysis of differential metabolites in *E. coli* stimulated by CPFX. Pathway enrichment significance is expressed as −log10(*p*-value) derived from hypergeometric tests against KEGG pathway annotations. Red dots indicate pathways with higher enrichment significance.

To systematically elucidate the functional consequences of these metabolic perturbations and infer underlying adaptive mechanisms, we performed KEGG pathway enrichment analysis on the identified differential metabolites for each antibiotic treatment. As depicted in Figure 3A–C, these analyses reveal the statistically significant enrichment of specific metabolic pathways, with the *x*-axis representing the enrichment significance as the −log(*p*-value) and the *y*-axis delineating the respective metabolic pathways. Highly significant enrichments are indicated by red points, while yellow points denote pathways with substantial, albeit less pronounced, enrichment.

In the OFLX-treated cohort, *E. coli* exhibited profound enrichment in pathways associated with purine metabolism, pyrimidine metabolism, and riboflavin metabolism (Figure 3A). This pattern suggests a substantial redirection of cellular resources towards the synthesis and turnover of nucleic acid precursors and critical cofactors.

The ENR-treated group displayed a similar, yet expanded, profile of pathway enrichment (Figure 3B). Here, significant enrichment was observed not only in purine and pyrimidine metabolism but also in the biosynthesis of pantothenate and coenzyme A (CoA), alongside riboflavin biosynthesis. This implies that ENR exposure triggers a robust adaptive response in *E. coli*, actively bolstering the production of essential nucleotides and cofactors—particularly CoA, which is central to energy metabolism, and riboflavin, a precursor for FAD—as a critical countermeasure to the metabolic challenges imposed by this fluoroquinolone.

Conversely, the CPFX-exposed group presented a distinct metabolic reconfiguration (Figure 3C). Pathways involved in pantothenate and coenzyme A biosynthesis were again notably enriched, but critically, this was accompanied by strong enrichment in the pentose phosphate pathway (PPP) and glutathione metabolism. This profile indicates a strategic redirection of carbon flux through the PPP to generate NADPH, which is essential for maintaining redox homeostasis, and a heightened engagement of the glutathione system, signifying a direct response to oxidative stress. Such coordinated pathway activation underscores *E. coli*’s attempt to mitigate the specific antibiotic pressure exerted by CPFX and preserve cellular integrity.

Collectively, these pathway enrichment analyses underscore that while all three fluoroquinolones exert their lethality through primary DNA damage, they elicit remarkably distinct and specific metabolic reconfigurations in *E. coli*. These varied adaptive responses not only illuminate the nuanced strategies bacteria deploy to cope with different antibiotic stressors but also highlight the drug-specific ways in which these agents perturb and ultimately compromise bacterial physiology.

### 3.4. Differential Metabolic Perturbations in Central Bacterial Networks

**Figure 4:**
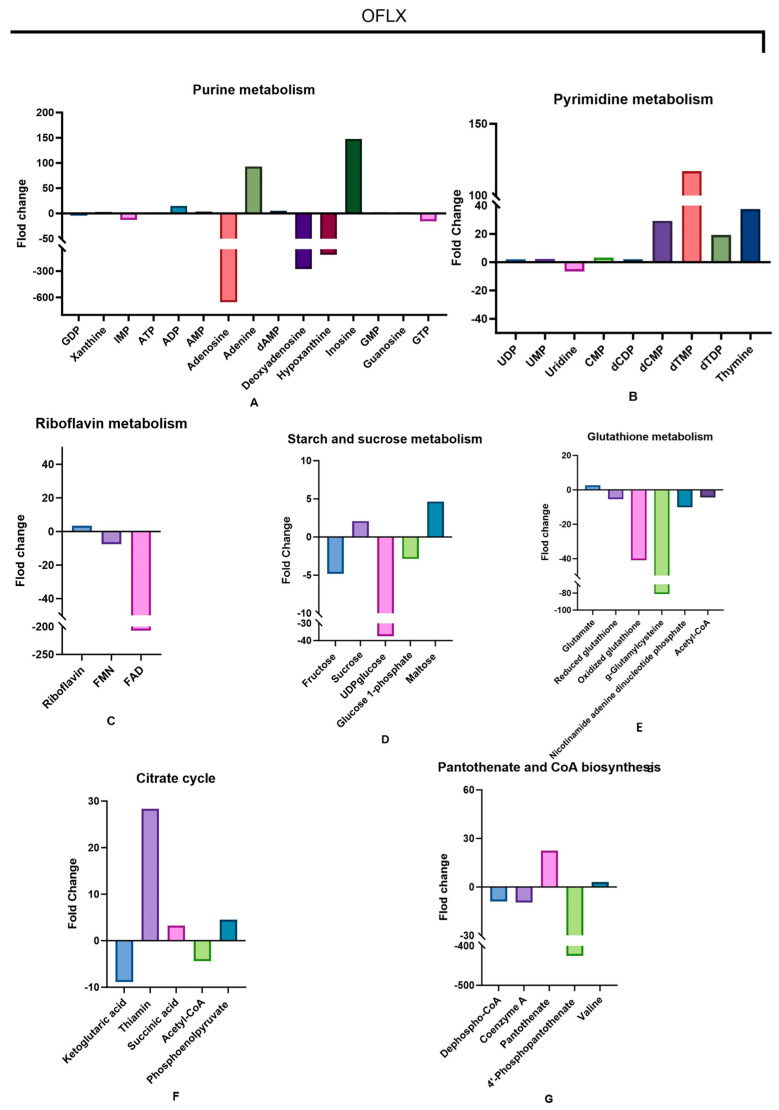
OFLX upregulation and downregulation of metabolites in key altered pathways. Extreme fold-changes reflect treated-cell concentrations approaching the detection limit relative to controls. Values are mean ± SD (n = 6). (**A**) Differential metabolites involved in purine metabolism, including GDP, xanthine, IMP, ATP, ADP, AMP, adenosine, adenine, dAMP, deoxyadenosine, hypoxanthine, inosine, GMP, guanosine, and GTP. (**B**) Differential metabolites involved in pyrimidine metabolism, including UDP, UMP, uridine, cMP, dCDP, dCMP, dTMP, dTDP, and thymine. (**C**) Differential metabolites involved in riboflavin metabolism, including riboflavin, FMN, and FAD. (**D**) Differential metabolites involved in starch and sucrose metabolism, including fructose, sucrose, UDP-glucose, glucose-1-phosphate, and maltose. (**E**) Differential metabolites involved in glutathione metabolism, including glutamate, reduced glutathione, oxidized glutathione, glutamylcysteine, nicotinamide adenine dinucleotide phosphate, and acetyl-CoA. (**F**) Differential metabolites involved in the citrate cycle (TCA cycle), including ketoglutaric acid, citrinin, succinic acid, acetyl-CoA, and phosphoenolpyruvate. (**G**) Differential metabolites involved in pantothenate and CoA biosynthesis, including dephospho-CoA, coenzyme A, pantothenate, 4′-phosphopantothenate, and valine.

**Figure 5:**
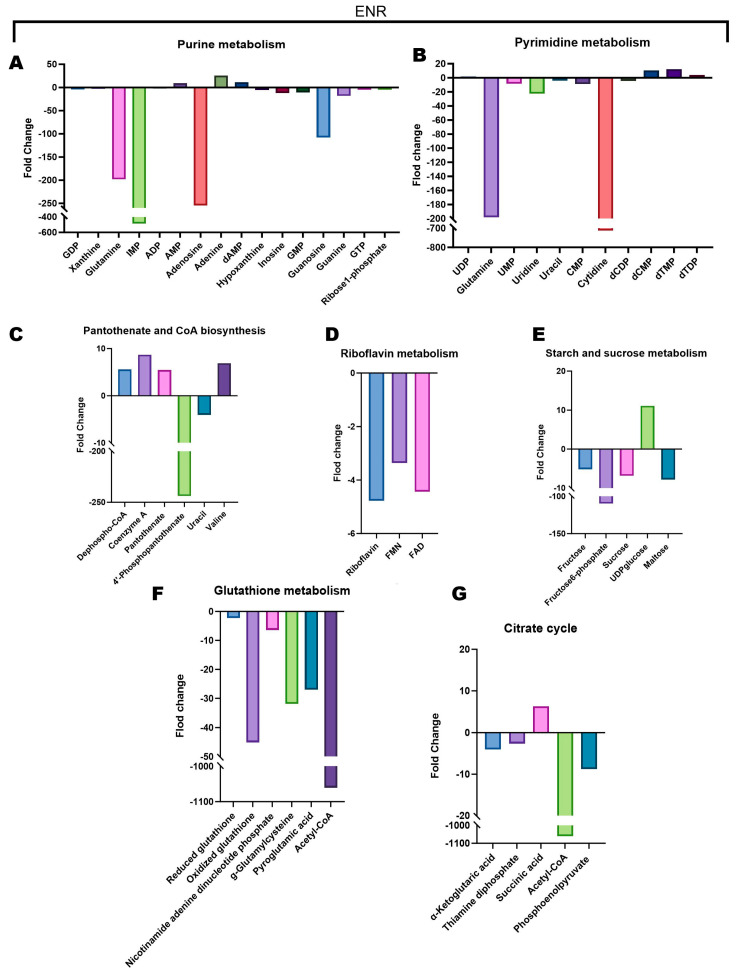
ENR upregulation and downregulation of metabolites in key altered pathways. Extreme fold-changes reflect treated-cell concentrations approaching the detection limit relative to controls. Values are mean ± SD (n = 6). (**A**) Differential metabolites involved in purine metabolism, including GDP, xanthine, glutamine, IMP, ADP, AMP, inosine, adenine, AMP, hypoxanthine, inosine, GMP, guanosine, GTP, and ribose-1-phosphate. (**B**) Differential metabolites involved in pyrimidine metabolism, including UDP, glutamine, UMP, uridine, uracil, CMP, cytidine, dCDP, dCMP, dTMP, and dTDP. (**C**) Differential metabolites involved in pantothenate and CoA biosynthesis, including dephospho-CoA, coenzyme A, pantothenate, 4′-phosphopantothenate, uracil, and valine. (**D**) Differential metabolites involved in riboflavin metabolism, including riboflavin, FMN, and FAD. (**E**) Differential metabolites involved in starch and sucrose metabolism, including fructose, fructose-6-phosphate, sucrose, UDP-glucose, and maltose. (**F**) Differential metabolites involved in glutathione metabolism, including reduced glutathione, oxidized glutathione, nicotinamide adenine dinucleotide phosphate, glutamylcysteine, pyroglutamic acid, and acetyl-CoA. (**G**) Differential metabolites involved in the citrate cycle (TCA cycle), including α-ketoglutaric acid, thiamine diphosphate, succinic acid, acetyl-CoA, and phosphoenolpyruvate.

**Figure 6:**
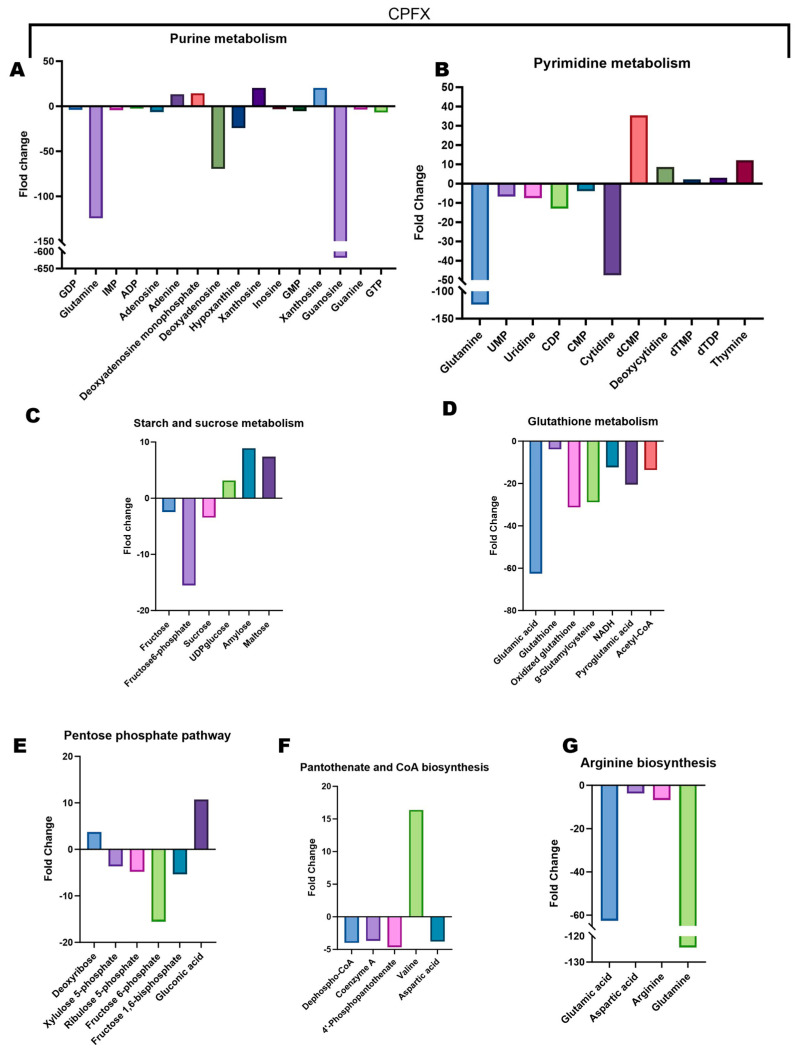
CPFX upregulation and downregulation of metabolites in key altered pathways. Extreme fold-changes reflect treated-cell concentrations approaching the detection limit relative to controls. Values are mean ± SD (n = 6). (**A**) Alterations in purine metabolism after CPFX exposure, showing substantial depletion of several purine-related intermediates, particularly glutamine, hypoxanthine, and guanosine, indicating severe disruption of nucleotide biosynthesis and salvage pathways. (**B**) Changes in pyrimidine metabolism following CPFX treatment. Significant fluctuations in glutamine, cytidine monophosphate (CMP), cytidine, and deoxycytidine monophosphate (dCMP) suggest extensive remodeling of pyrimidine nucleotide metabolism. (**C**) Differential abundance of metabolites involved in starch and sucrose metabolism. CPFX treatment resulted in decreased fructose-6-phosphate and sucrose levels, while amylose and maltose accumulated, indicating altered carbohydrate utilization and energy metabolism. (**D**) Changes in glutathione metabolism in response to CPFX treatment. Marked reductions in glutamic acid, oxidized glutathione, and glutarylmethylamine imply impaired redox homeostasis and enhanced oxidative stress. Fold changes were calculated relative to untreated controls. (**E**) Differential metabolites involved in the pentose phosphate pathway, including deoxyribose, xylulose-5-phosphate, ribose-5-phosphate, fructose-6-phosphate, fructose-1,6-bisphosphate, and gluconic acid. (**F**) Differential metabolites involved in pantothenate and CoA biosynthesis, including dephospho-CoA, coenzyme A, 4′-phosphopantothenate, valine, and aspartic acid. (**G**) Differential metabolites involved in arginine biosynthesis, including glutamic acid, aspartic acid, arginine, and glutamine.

To further dissect the intricate metabolic adaptations of *E. coli* under varying fluoroquinolone pressures, we conducted a comprehensive quantitative analysis of relative concentration changes in key metabolites across multiple metabolic pathways. Bar charts (Figure 4, Figure 5 and Figure 6) meticulously detail the impact of each antibiotic treatment on specific metabolite levels.

OFLX treatment induced notable alterations across several pathways. Within purine metabolism, we observed a pronounced downregulation of adenosine, accompanied by discernible reductions in deoxyadenosine and hypoxanthine. Conversely, metabolites such as adenine and inosine demonstrated an upward trend in their levels (Figure 4A), suggesting a redirection or imbalance in purine interconversion. Pyrimidine metabolism exhibited elevated concentrations of dCMP, thymine, and dTMP (Figure 4B), indicative of perturbed pyrimidine nucleotide synthesis or degradation. A striking perturbation in riboflavin metabolism was evident, with FAD concentration experiencing a substantial 207.3-fold decrease (Figure 4C), signaling a severe deficiency in this critical redox cofactor. In the context of starch and sucrose metabolism, both fructose and UDP-glucose were significantly downregulated (Figure 4D), implying impaired carbohydrate catabolism or cell wall precursor synthesis. Glutathione metabolism was impacted, as evidenced by a marked decrease in the concentrations of both oxidized glutathione and γ-glutamylcysteine (Figure 4E), pointing to compromised antioxidant defenses. Furthermore, the tricarboxylic acid (TCA) cycle revealed significantly elevated levels of thiamine and phosphoenolpyruvate, contrasted by reduced concentrations of α-ketoglutarate and acetyl-CoA (Figure 4F), suggesting a potential bottleneck or re-routing of carbon flux within central metabolism. Finally, within the pantothenate and coenzyme A biosynthesis pathway, a significant decline in 4′-phosphopantetheine concentration was noted (Figure 4G), which could impact CoA availability for numerous metabolic processes.

ENR treatment elicited a dramatic and distinct metabolic shift. Within the purine metabolism pathway, IMP concentration plummeted by an astounding 486.1-fold, alongside a decrease in guanosine levels (Figure 5A), indicating severe disruption of purine nucleotide pools. In pyrimidine metabolism, both glutamine and cytidine experienced substantial downregulation (Figure 5B), further highlighting an impact on nucleotide synthesis. The pantothenate and coenzyme A biosynthesis pathway again showed a significant reduction in 4′-phosphopantetheine levels (Figure 5C), consistent with OFLX’s effect but potentially through different mechanisms. Riboflavin metabolism was severely compromised, with both riboflavin and FAD levels exhibiting significant downregulation (Figure 5D), mirroring the profound FAD depletion seen with OFLX. Pertaining to starch and sucrose metabolism, fructose-6-phosphate concentration was precipitously reduced by 109.7-fold, while UDP-glucose levels showed a slight increase (Figure 5E), suggesting a complex interplay in carbohydrate utilization. Notably, glutathione metabolism was profoundly altered, indicated by an extraordinary 1060.7-fold drop in acetyl-CoA (Figure 5F), a central metabolite whose depletion would have widespread consequences for energy and biosynthetic pathways. Consistently, acetyl-CoA levels were also diminished within the TCA cycle (Figure 5G), underscoring a broad impairment of energy-generating processes.

CPFX exposure induced a unique metabolic profile. The purine metabolism pathway displayed a subtle downregulation of glutamine, deoxyadenosine, and hypoxanthine, concurrently with a substantial 618.4-fold decrease in guanosine levels (Figure 6A). In pyrimidine metabolism, despite a minor elevation in dCMP, both glutamine and cytidine underwent significant reductions (Figure 6B). The starch and sucrose metabolism pathway demonstrated increases in maltose and starch, juxtaposed with a marked decline in fructose-6-phosphate levels (Figure 6C), indicating altered carbohydrate utilization and storage. Within glutathione metabolism, both oxidized glutathione and glutamate were notably downregulated (Figure 6D), signifying a direct impact on redox homeostasis and stress response. Additionally, in the pentose phosphate pathway, we observed a decrease in xylulose-5-phosphate levels, paralleled by an increase in deoxyribose (Figure 6E), suggesting a shift in sugar phosphate metabolism. The pantothenate and coenzyme A biosynthesis pathway exhibited a significant elevation in valine levels (Figure 6F), which might reflect a compensatory mechanism or altered amino acid turnover. Curiously, in the arginine biosynthesis pathway, while glutamate and glutamine concentrations significantly decreased, arginine levels themselves showed only an insignificantly modest downregulation (Figure 6G), indicating potential robustness or alternative synthesis routes for arginine under CPFX stress despite precursors being depleted. Notably, the exceptionally large fold-changes observed for certain metabolites, such as the 1060.7-fold decrease in acetyl-CoA under ENR treatment, likely reflect concentrations in treated cells that approached the lower limit of detection relative to control levels. These extreme values underscore the profound impact of fluoroquinolone stress on core metabolic pools.

### 3.5. Fluoroquinolones Drive ROS Production and Redox Imbalance in Bacteria

**Figure 7:**
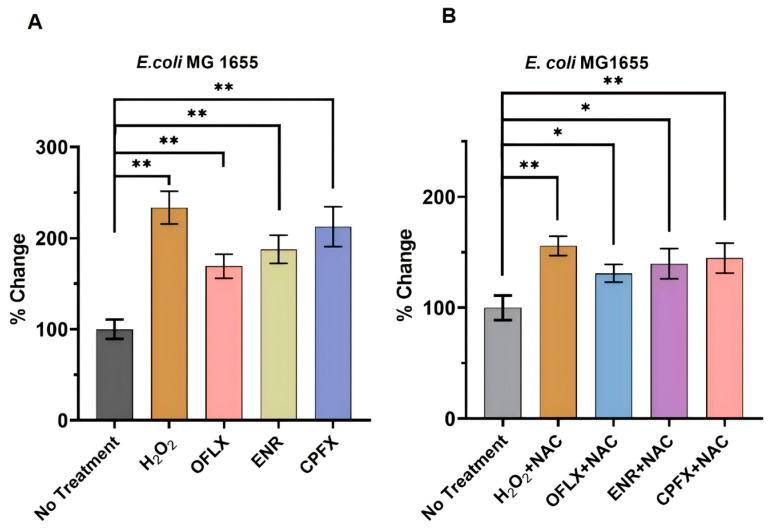
Intracellular accumulation of ROS in bacteria induced by fluoroquinolone antibiotics. (**A**) ROS accumulation dynamics upon antimicrobial stimulation. (**B**) Repercussions of ROS inhibitors on intracellular ROS levels. Values are mean ± SD (n = 6). * *p* < 0.05, ** *p* < 0.01 vs. control (ANOVA with Tukey’s test).

In the present investigation, we systematically examined the cascade of metabolic derangements triggered by three clinically prevalent fluoroquinolone antibiotics-OFLX, ENR, and CPFX-in *E. coli*. Our investigation further revealed a significant upregulation of intracellular reactive oxygen species (ROS) in *E. coli* MG1655 following treatment with these antimicrobials (Figure 7A). Untreated controls established a baseline ROS level. As a robust positive control, hydrogen peroxide (H_2_O_2_) dramatically elevated ROS levels to 233.60 ± 17.90% of the untreated control, confirming the sensitivity of our experimental system for detecting oxidative stress. Crucially, all three tested fluoroquinolone antimicrobials elicited substantial and statistically significant increases in intracellular ROS compared to the untreated control group (*p* < 0.01). Ciprofloxacin (CPFX) led to the highest ROS levels, reaching 212.65 ± 21.94% of baseline, followed by enrofloxacin (ENR) at 187.75 ± 15.43%, and ofloxacin (OFLX) at 169.17 ± 13.22%. These findings collectively underscore that fluoroquinolones are potent inducers of oxidative stress in *E. coli* MG1655 under in vitro conditions, manifested by a pronounced accumulation of intracellular ROS. The observed moderate-to-high ROS generation, particularly by CPFX and ENR, suggests that the production of reactive oxygen species plays a pivotal role in their antimicrobial mechanisms, contributing significantly to their bactericidal effects and associated cellular toxicity. This evidence strongly supports the notion that a fluoroquinolone-induced ROS surge is an important component of their antibacterial action.

To further investigate the causal link between elevated ROS levels and antibiotic action, we conducted experiments designed to inhibit ROS production using the known antioxidant *N*-acetylcysteine (NAC) in co-treatment with each antibiotic. The introduction of NAC significantly mitigated the increase in ROS levels induced by all three fluoroquinolones. Specifically, ROS levels, when co-treated with NAC, were reduced to 144.75 ± 13.51% for CPFX, 139.73 ± 13.70% for ENR, and 131.05 ± 8.03% for OFLX (Figure 7B), compared to their respective antibiotic-only treatments. These findings reinforce the conclusion that fluoroquinolone administration effectively elevates ROS levels in bacterial cells, an effect that can be substantially attenuated through the application of ROS scavengers, thereby highlighting the therapeutic potential of targeting oxidative stress pathways.

### 3.6. Fluoroquinolones Drive Oxidative Nucleic Acid Lesions

ROS are well-established mediators of nucleic acid damage, inflicting characteristic oxidative lesions through diverse chemical mechanisms. Among the most critical markers of such damage are 8-hydroxydeoxyguanosine (8-OHdG) and 8-hydroxyguanosine (8-OHG). These adducts are signature products formed when highly reactive species, particularly hydroxyl radicals and superoxide anions, nucleophilically attack the C8 position of guanine residues embedded within DNA and RNA backbones, respectively. As canonical indicators of nucleic acid oxidative burden, the quantitative accumulation of 8-OHdG and 8-OHG provides direct measures of the cellular oxidative stress endured.

**Figure 8:**
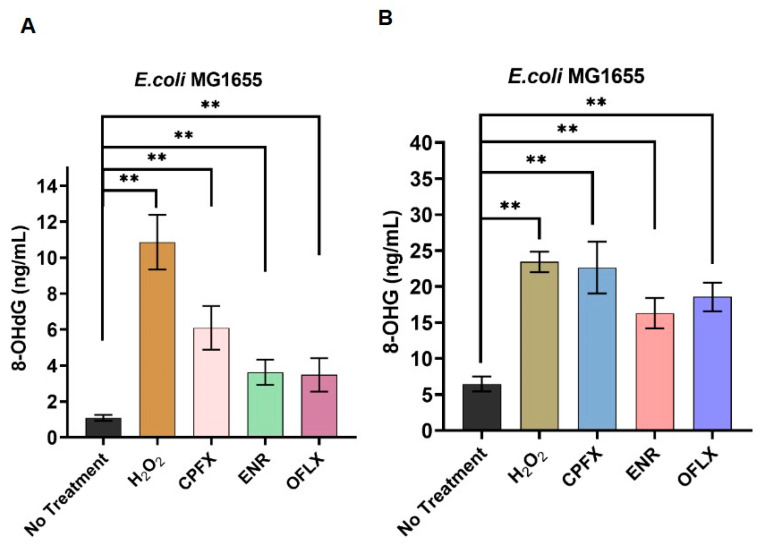
Intracellular DNA/RNA damage induced by Fluoroquinolone antibiotics. (**A**) Impact of antibacterial agents on the intracellular DNA levels of 8-OHdG. (**B**) Impact of antibacterial agents on the intracellular RNA levels of 8-OHG.Values are mean ± SD (n = 6).

In the present study, we systematically evaluated the capacity of three structurally distinct fluoroquinolone agents—OFLX, ENR, and CPFX-to promote oxidative damage to nucleic acids in *E. coli* MG1655 by quantifying intracellular 8-OHdG and 8-OHG accumulation. Our data consistently revealed that all fluoroquinolone-treated cohorts exhibited significantly elevated 8-OHdG levels relative to vehicle-treated controls. The untreated control group displayed a basal 8-OHdG concentration of 1.09 ± 0.16 ng/mL. In stark contrast, the hydrogen peroxide (H_2_O_2_) positive control group showed a substantial increase to 10.86 ± 1.52 ng/mL, representing an approximate 9.96-fold elevation. Among the fluoroquinolones, CPFX treatment induced the highest 8-OHdG accumulation, reaching 6.10 ± 1.21 ng/mL (approximate 5.59-fold increase), while OFLX and ENR treatments resulted in 3.49 ± 0.93 ng/mL (approximate 3.20-fold increase) and 3.63 ± 0.71 ng/mL (approximate 3.33-fold increase), respectively (Figure 8A). Notably, all fluoroquinolone treatments demonstrated statistically significant increases compared to the untreated control (*p* < 0.01). Analysis of 8-OHG abundance disclosed a parallel and equally pronounced pattern of oxidative damage. The untreated control group maintained an 8-OHG level of 6.48 ± 1.04 ng/mL. The H_2_O_2_ positive control group exhibited a significant elevation to 23.4 ± 1.42 ng/mL, corresponding to an approximate 3.61-fold increase. All fluoroquinolone treatment conditions triggered substantial increments in 8-OHG levels when compared against untreated controls. CPFX treatment yielded 22.63 ± 3.60 ng/mL (approximate 3.49-fold increase), followed by OFLX at 18.58 ± 1.99 ng/mL (approximate 2.86-fold increase), and ENR at 16.30 ± 2.09 ng/mL (approximate 2.52-fold increase) (Figure 8B). These elevations were also statistically significant compared to the untreated control (*p* < 0.01).

Collectively, these findings underscore a fundamental and previously underappreciated mechanism by which fluoroquinolone antibiotics exert their bactericidal effects. Beyond their canonical inhibition of bacterial topoisomerases, these agents concurrently orchestrate a heightened oxidative assault on bacterial nucleic acids. The marked elevation in both 8-OHdG and 8-OHG levels corroborates our earlier demonstrations of elevated intracellular ROS, strongly suggesting that the bactericidal efficacy of fluoroquinolones is intrinsically linked to oxidative nucleic acid damage. This synergistic interplay, wherein ROS accumulation precipitates multi-faceted oxidative lesions that contribute to both metabolic dysfunction and genomic integrity compromise, likely constitutes a central pillar of fluoroquinolone-mediated bacterial killing.

## 4. Discussion

Our study provides a comprehensive, high-resolution dissection of the intricate metabolic landscape perturbed by three distinct fluoroquinolone antibiotics—ofloxacin (OFLX), enrofloxacin (ENR), and ciprofloxacin (CPFX)-in *Escherichia coli* MG1655. Critically, we demonstrate that beyond their canonical primary target of DNA gyrase and topoisomerase IV, fluoroquinolones orchestrate drug-specific metabolic reconfigurations that culminate in substantial reactive oxygen species (ROS) production and widespread oxidative damage to both DNA and RNA. This multifaceted mechanism fundamentally expands our understanding of fluoroquinolone bactericidal action, highlighting the importance of secondary metabolic liabilities in their efficacy.

Initially, we rigorously defined the bactericidal concentrations of OFLX, ENR, and CPFX (2xMIC) that achieved rapid and potent killing of *E. coli* MG1655 within 1.5 h (Section 3.1). A crucial aspect of this initial characterization was the scanning electron microscopy (SEM) analysis, which revealed that at these bactericidal concentrations, bacterial cells maintained their intact morphology, exhibiting no overt signs of membrane damage or lysis. While SEM cannot directly prove metabolic integrity, this observation remains paramount for the subsequent metabolomic analyses, as the absence of visible membrane disruption supports that the intracellular metabolite profiles observed are genuine reflections of metabolic perturbation rather than artifacts of passive metabolite leakage from compromised cell membranes. This meticulous validation of experimental conditions establishes a robust foundation for the reliability and physiological relevance of our metabolomic data, allowing us to confidently attribute changes in metabolite abundance to active cellular responses and drug-induced metabolic shifts rather than non-specific cellular disintegration [28].

The core of our investigation lies in the revelation of distinct metabolic reconfigurations induced by each fluoroquinolone (Section 3.2 and Section 3.3). Using UPLC–Q Exactive Orbitrap–MS and hierarchical clustering, we uncovered significant, yet often divergent, perturbations across the intracellular metabolome. This finding challenges a simplified view of fluoroquinolone action, demonstrating that despite sharing a common primary target (DNA topoisomerases), the downstream metabolic consequences are far from uniform. OFLX treatment elicited a complex response marked by upregulation of crucial coenzymes, amino acids, and nucleotides, alongside a profound 207.3-fold decrease in FAD, suggesting an intensified demand for nucleic acid precursors and critical redox cofactors, possibly driven by a need for DNA repair and cellular maintenance, but simultaneously indicating a severe deficiency in redox buffering [29,30]. ENR, while also showing some compensatory cofactor and amino acid upregulation, induced an extraordinary 486.1-fold plummet in IMP and a staggering 1060.7-fold drop in acetyl-CoA, indicating a severe disruption of purine nucleotide pools and a broad impairment of central energy metabolism. In stark contrast, CPFX exhibited a more generalized metabolic collapse, with fewer compensatory upregulation signals and a pronounced downregulation of core energy metabolism metabolites, critical coenzymes (e.g., acetyl-CoA, NADH), and crucial antioxidant compounds like reduced glutathione. These findings are consistent with previous reports highlighting the varied physiological impacts of different fluoroquinolones [31].

These divergent responses may stem from the drugs’ distinct structural properties; for instance, the higher lipophilicity of CPFX and its specific affinity for DNA gyrase likely trigger a more intense ‘bioenergetic catastrophe’ compared to the veterinary agent ENR.

Pathway enrichment analysis (Section 3.3) further underscored these drug-specific metabolic strategies. OFLX treatment prominently enriched purine, pyrimidine, and riboflavin metabolism, directly connecting to the synthesis and turnover of nucleic acid building blocks and redox cofactors. ENR broadened this response to include pantothenate and coenzyme A biosynthesis, suggesting a more robust adaptive response involving essential nucleotides and central energy metabolism cofactors. Most strikingly, CPFX uniquely enriched the pentose phosphate pathway (PPP) and glutathione metabolism, alongside pantothenate and CoA biosynthesis. The PPP is a critical source of NADPH, vital for maintaining redox homeostasis, while the glutathione system is a primary defense against oxidative stress [32,33]. This specific enrichment with CPFX provided a strong mechanistic hint toward its potent induction of oxidative stress, a hypothesis we subsequently investigated.

These results suggested that CPFX might exert more significant inhibitory effects on the bacterial antioxidant system compared to OFLX and ENR. The observed divergent metabolic responses among OFLX, ENR, and CPFX may be attributed to their distinct structural properties and pharmacokinetic profiles. For instance, while all three agents target DNA topoisomerases, their varying affinities for DNA gyrase versus topoisomerase IV, and their different lipophilicity levels, likely dictate the intensity of the downstream ‘bioenergetic catastrophe’. Specifically, CPFX, a second-generation fluoroquinolone with potent activity in humans, exhibited the most severe suppression of antioxidant systems, whereas the veterinary-specific drug ENR showed a unique disruption of the purine pool (IMP). These nuances suggest that fluoroquinolone lethality is not a monolithic process but is finely tuned by the specific chemical scaffold of the drug.

**Figure 9:**
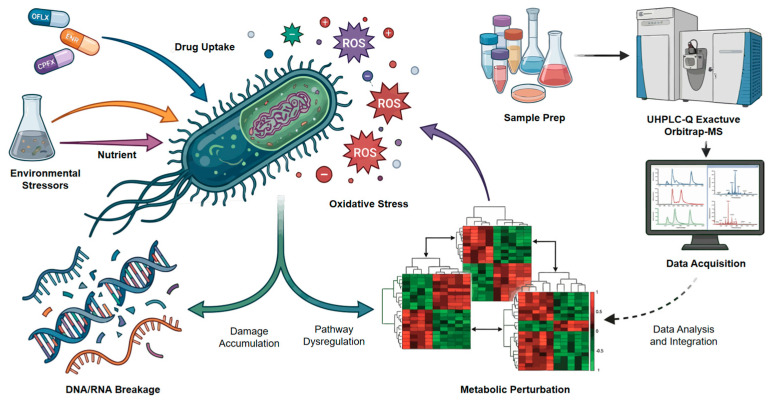
Fluoroquinolone antibiotics induce metabolic perturbations that lead to cellular damage.

The quantitative analysis of relative concentration changes in key metabolites (Section 3.4) provided granular evidence for these pathway-level insights. Figure 9 further illustrates that fluoroquinolone antibiotics induce metabolic perturbations that lead to cellular damage. For instance, the dramatic FAD depletion by OFLX and riboflavin/FAD depletion by ENR underscore severe compromises in cellular redox capacity. The profound reduction in IMP by ENR, a central precursor for purine nucleotides, indicates a severe bottleneck in nucleic acid synthesis, potentially contributing to its bactericidal effect. CPFX’s impact on carbohydrate metabolism (e.g., fructose-6-phosphate) and core energy cofactors (e.g., GTP, ADP, acetyl-CoA) painted a picture of widespread energy deficit and inability to mount robust metabolic responses. This fine-grained resolution of metabolite changes reveals how each fluoroquinolone, through its unique metabolic fingerprint, creates distinct vulnerabilities within the bacterial cell.

The direct link between fluoroquinolone treatment and ROS production and redox imbalance was definitively established in Section 3.5. All three fluoroquinolones significantly increased intracellular ROS levels in *E. coli* MG1655, with CPFX being the most potent inducer, followed by ENR and OFLX. This finding is consistent with a growing body of literature demonstrating that many bactericidal antibiotics, including fluoroquinolones, induce oxidative stress as a major component of their killing mechanism [6,34]. The subsequent experiment with the antioxidant N-acetylcysteine (NAC) confirmed the causal role of ROS, as NAC significantly mitigated the fluoroquinolone-induced ROS surge. This quantitative evidence strongly supports the notion that fluoroquinolones do not merely inhibit DNA replication but actively drive a deleterious oxidative cascade within the bacterial cell. The differential potency of ROS induction among the three fluoroquinolones (CPFX > ENR > OFLX) directly correlates with the severity of metabolic disruption observed in the earlier metabolomic profiles, particularly CPFX’s pronounced impact on antioxidant systems and activation of the PPP, a primary response to oxidative stress.

Finally, we provided compelling evidence that this fluoroquinolone-induced ROS production translates into oxidative nucleic acid lesions (Section 3.6). The significant elevation of 8-hydroxydeoxyguanosine (8-OHdG) and 8-hydroxyguanosine (8-OHG), canonical markers of oxidative DNA and RNA damage, respectively, confirmed that ROS generated by fluoroquinolones inflict tangible damage on genetic material. Again, CPFX, the strongest ROS inducer, led to the highest accumulation of both 8-OHdG and 8-OHG, reinforcing a clear mechanistic link between ROS production and nucleic acid damage [35]. This is a critical finding, as it delineates a secondary, oxidative mechanism of nucleic acid damage that operates in parallel to, or perhaps in synergy with, the direct topoisomerase inhibition by fluoroquinolones. While direct DNA damage from topoisomerase inhibition is well-established, the contribution of oxidative damage, exacerbated by metabolic dysregulation, has been less comprehensively understood in the context of drug-specific effects [36].

Collectively, our study paints a nuanced picture of fluoroquinolone action. We demonstrate that these antibiotics are not simply DNA gyrase inhibitors; rather, they are potent orchestrators of profound and distinct metabolic reconfigurations within *E. coli*. These metabolic shifts lead to varying degrees of oxidative stress, which, in turn, cause significant oxidative damage to bacterial nucleic acids. The drug-specific nature of these metabolic perturbations and their subsequent impact on ROS generation and oxidative lesions offers critical insights into the differential efficacy and potential for resistance development among various fluoroquinolones. This refined understanding opens new avenues for therapeutic strategies, including the development of combination therapies that exploit specific metabolic vulnerabilities or enhance ROS production, ultimately aiming to improve antimicrobial efficacy and overcome drug resistance. Further studies, perhaps involving genetic manipulation of key metabolic pathways or in vivo models, could further delineate the precise contribution of each metabolic perturbation to overall bacterial killing and drug susceptibility.

Several limitations should be noted. Our study employed a single time point (1 h) and an in vitro model. Future work using in vivo models and dynamic time-course analysis is needed to fully capture the evolution of metabolic collapse.

## 5. Conclusions

Despite the insights provided by this study, several limitations should be acknowledged. First, our metabolomic profiling was conducted at a single bactericidal concentration (2 × MIC) and a fixed time point (1 h), which may not capture the full temporal dynamics of the metabolic collapse. Second, this study was performed in vitro using *E. coli* MG1655 as a model; future research employing in vivo infection models and clinical isolates is necessary to validate the translational relevance of these metabolic adjuvants. Nonetheless, our findings establish a robust metabolic atlas for understanding fluoroquinolone-induced bacterial demise.

In summary, this study provides a systematic characterization of the metabolic perturbations induced by three representative fluoroquinolones—ofloxacin, enrofloxacin, and ciprofloxacin—in *Escherichia coli*. Despite sharing a common primary target, these antibiotics elicited both conserved and drug-specific metabolic responses, particularly affecting nucleotide biosynthesis, central carbon metabolism, and redox homeostasis. Notably, all treatments were associated with elevated intracellular reactive oxygen species (ROS) levels and increased oxidative damage to DNA and RNA, as evidenced by the accumulation of 8-OHdG and 8-OHG. These findings support a model in which fluoroquinolone-induced metabolic reprogramming is closely linked to oxidative stress and contributes to bacterial cell damage. The observed divergence in metabolic responses among different fluoroquinolones further highlights the importance of drug-specific physiological effects. Overall, this work expands the current understanding of fluoroquinolone bactericidal activity from a metabolic perspective and provides a foundation for the development of potential metabolic adjuvant strategies to enhance antibiotic efficacy.

## Data Availability

The data presented in this study are available within the article. Additional data supporting the findings of this study are available from the corresponding author upon reasonable request.

## Abbreviations

The following abbreviations are used in this manuscript:

FQs	Fluoroquinolones
OFLX	Ofloxacin
CPFX	Ciprofloxacin
UPLC–Orbitrap–MS	Ultra-performance liquid chromatography–Orbitrap mass spectrometry
HILIC	Hydrophilic interaction liquid chromatography
8-OHdG	8-hydroxydeoxyguanosine
ENR	Enrofloxacin
